# *Helicobacter pylori* upregulates circPGD and promotes development of gastric cancer

**DOI:** 10.1007/s00432-023-05537-w

**Published:** 2024-02-26

**Authors:** Wenjun Zhao, Zhendong Yao, Jia Cao, Yun Liu, Linqi Zhu, Boneng Mao, Feilun Cui, Shihe Shao

**Affiliations:** 1 The Affiliated Yixing Hospital of Jiangsu University, Wuxi, 214200 Jiangsu China; 2 Urology Department, The Affiliated Taizhou Second People’s Hospital of Yangzhou University, Taizhou, 225500 Jiangsu China; 3 School of Medicine, Shanghai East Hospital, Tongji University, Shanghai, 200120 China; 4 Jiangsu University School of Medicine, Zhenjiang, 212013 Jiangsu China

**Keywords:** *Helicobacter pylori*, circRNAs, Gastric cancer, Inflammation, Apoptosis

## Abstract

**Purpose:**

*Helicobacter pylori* (*H. pylori*) has unique biochemical traits and pathogenic mechanisms, which make it a substantial cause of gastrointestinal cancers. Circular RNAs (circRNAs) have concurrently been identified as an important participating factor in the pathophysiology of several different cancers. However, the underlying processes and putative interactions between *H. pylori* and circRNAs have received very little attention. To address this issue, we explored the interaction between *H. pylori* and circRNAs to investigate how they might jointly contribute to the occurrence and development of gastric cancer.

**Methods:**

Changes in circPGD expression in *H. pylori* were detected using qRT-PCR. Cell proliferation and migration changes were assayed by colony formation, the CCK-8 assay and the transwell assay. Apoptosis was measured by flow cytometry. Western blot was conducted to detect changes in cell migration, apoptosis, proliferation and inflammation-associated proteins. QRT-PCR was used to measure changes in circPGD and inflammation-associated factors.

**Results:**

We found that *H. pylori* induced increased circPGD expression in infected human cells and facilitated gastric cancer progression in three ways by promoting cell proliferation and migration, enhancing the inflammatory response, and inhibiting apoptosis.

**Conclusions:**

CircPGD appears to play a role in *H. pylori*-related gastric cancer and may thus be a viable, novel target for therapeutic intervention.

**Supplementary Information:**

The online version contains supplementary material available at 10.1007/s00432-023-05537-w.

## Introduction

Gastric cancer, also known as stomach cancer, is a relatively common malignancy, being the third most common cause of cancer-related deaths worldwide (Lu et al. 2022; Ma et al. 2022). With a relative five-year survival rate below 30% in its advanced stage, it has a poor prognosis and represents a substantial socio-medical burden that remains to be addressed (Yu et al. 2023). Research on the disease has linked numerous non-coding RNAs to its onset and malignant spread. These non-coding RNAs are currently considered highly promising biomarkers for determining the early risk of individuals with cancer, their therapeutic outlook, and likely survival (Niu et al. 2023). Among the different types of non-coding RNAs, circular RNAs (circRNAs) have emerged as a unique class distinguished by a covalently closed circular structure that confers resistance to digestion by RNase R. CircRNAs have been found to be highly expressed in several tumor tissues, including gastric cancer (Zhou et al. 2023; Liu et al. 2023). For instance, the expression of the circMTHFD2L-encoded CM-248aa protein is reportedly markedly decreased in gastric cancer. Research on this protein has discovered that AKT, extracellular signal-regulated kinase, and P65 all become dephosphorylated as a result of CM-248aa's binding to the acidic structural domain of the SET nuclear oncogene, which functions as an endogenous inhibitor of SET-protein phosphatase 2A. Thus, CM-248aa rather than circMTHFD2L inhibits the development of gastric cancer (Liu et al. 2023). Furthermore, circTDRD3 has been identified to be highly expressed in gastric cancer tissues, promoting the growth and spread of gastric cancer cells both in vivo and in vitro. These effects are accomplished via the transcription factor ATF891 modulating the miR-2b/ITGA4 axis and the AKT signaling pathway (Zhou et al. 2023).

Marshall and Warren were the first to discover *Helicobacter pylori* (*H. pylori*), a microaerobic *Campylobacter* that predominantly resides in the stomach and causes gastritis and peptic ulcers (Nabavi-Rad et al. 2023; Malfertheiner et al. 2023). In addition to secreting proteases, cytotoxin-associated protein (CagA), and vacuolar cytotoxin A (VacA), *H. pylori* features several other virulent characteristics. These aid in the breakdown of the gastric mucosa, the initiation of inflammatory signaling in both immunological and epithelial cells, and the recruitment of cytokines and chemokines (including IL-1, IL-6, IL-11, IL-12, IL-17, and TNFα) in the chronic inflammation phase of Wang et al. (2014), Lee et al. 2016). Inflammation serves as a pivotal marker of tumorigenesis (Yu et al. 2023), and numerous studies have shown that inflammatory responses are magnified at the tumor site, promote the spread of cancer. This amplification process is frequently induced by interaction between various immune and inflammatory responses (Domínguez-Martínez et al. 2023; Zhang et al. 2022). As a result of such interactions occurring between the pathogen, immunity, and carcinogenesis, World Health Organization has designated *H. pylori* as a Group 1 carcinogen (Li et al. 2019; Wang et al. 2019). Although a few studies have investigated the involvement of certain circRNAs in facilitating the pathogenesis and progression of *H. pylori*-associated gastric cancer, further research must still be conducted to explain the underlying mechanistic processes at play.

Therefore, utilizing the existing body of research already showing that hsa_circ_0009735 (also known as circPGD) may promote the progression of gastric cancer(Liu et al. 2022), the current study used knockdown and overexpression strategies to delve deeper into the involvement presented herein, in *H. pylori*-related gastric cancer. According to the research presented herein, *H. pylori* promotes the growth of gastric cancer by increasing the expression of circPGD, which in turn affects cellular migration, apoptosis proliferation, and inflammation. These findings are expected to open the door to cutting-edge therapeutic strategies that specifically target gastric cancer linked to *H. pylori*.

## Methods

### Patients and samples

A total of 66 tissue samples were obtained from Shanghai Oriental Hospital to use in the study. These samples included gastroscopic tissues obtained from individuals divided into three groups: healthy persons, those positive for *H. pylori* infection, and those with gastric cancer.

### RNA extraction and real-time quantitative PCR (qRT–PCR)

TRIzol reagent (Vazyme, Nanjing, China) was used to extract total RNA from gastric tissue and cell samples, after which reverse transcription reagents (Vazyme, Nanjing, China) were used to convert the extracted RNA into complementary DNA (cDNA). QRT-PCR was then performed to measure the expression levels of circRNAs and messenger RNA (mRNA), with the housekeeping gene GAPDH serving as an internal reference. The primer sequence used can be found in additional file 1: Table S1.

### Cell culture and *H. pylori* infection

*Helicobacter pylori* standard strain 26695, the human gastric cancer cell line HGC-27, and the normal human gastric epithelial cell line GES-1 were received by our lab in frozen form. *H. pylori* 26695 was grown on Columbia blood plates under microaerobic conditions (5% O_2_, 10% CO_2_, and 85% N_2_) for 72 h. The GES-1 and HGC-27 were grown in DMEM with 10% fetal bovine serum (Gibco, Invitrogen, Waltham, MA, USA) in a humid incubator (5% CO_2_, 37°C).

### Cell transfection

GenePharma Corporation (Shanghai, China) provided 125 nM of short interfering RNAs (siRNAs), targeting circPGD and the matching negative controls (NCs), The pcicR-3.0 plasmid was cloned to contain the whole length of circPGD (China's Asia-Vector Biotechnology, Shanghai, China). Lipofectamine 3000 transfection reagent (Invitrogen, USA) was used to transfect the siRNA and plasmid.

### Western blotting analysis and antibodies

Using radio-immunoprecipitation assay (RIPA) lysis buffer (Beyotime Biotechnology, Shanghai, China) supplemented with phenyl-methane-sulfonyl fluoride (PMSF), the total cellular proteins were isolated from the samples. The cell lysate was extracted, and subjected to 13,000*g* of centrifugation for 30 min at 4 °C. Then, the protein samples were electrophoresed on 12% SDS polyacrylamide gels to separate them. The resulting protein bands were then transferred onto polyvinylidene difluoride (PVDF) membranes (Millipore, MA,USA). Following blocked for 2 h at room temperature, the membranes were incubated overnight with the primary antibodies (additional file 2: Table S2) at 4 °C. The membranes were subsequently incubated with the secondary diluted antibody (1:2000) for an hour at room temperature prior to being exposed to enhanced chemiluminescence (ECL)using an ECL system (Image Quant LAS 4000 mini, Pittsburgh, PA, USA) for imaging.

### Cell proliferation and colony-formation assay

The Cell Counting Kit-8 (CCK8) (Vazyme, Nanjing, China) was used to measure cell proliferation after the cells were harvested and dispensed into 96-well plate (at 1000 cells per well). The cells were seeded into a 6-well plate and incubated for 12 days at 5% CO_2_, 37 °C, fixed in 4% paraformaldehyde, and stained with 0.1% crystal violet.

### Transwell migration assay

Cells were collected and inoculated in 24-well plate (5 × 10^5^/well) with serum-free medium in the upper chamber and 8-μm pore size uncoated membrane inserts (Corning, NY, USA) in the lower chamber. The lower chamber contained 10% FBS as a chemical elicitor. After 24 h, the cells were fixed in 4% paraformaldehyde, stained in 0.1% crystal violet, and the migrating cells were counted.

### RNA fluorescence in situ hybridization (RNA-FISH)

After being plated onto the glass coverslips, cells were subjected to further culture. Gene Pharma (Suzhou, China) designed and synthesized a Cy3-coupled circPGD probe, and operated according to manufacturer's instructions.

### Urease activity detection

The bacteria to be tested were inserted into the testing reagent. When the reagent turned red, it was taken to indicate urease positivity. If it remained yellow or the red disappeared rapidly, it was recorded as urease-negative.

### Apoptosis assay

The harvested cells were rinsed by pre-chilled PBS, followed by resuspension with 1× binding buffer. An apoptosis kit was purchased (Vazyme, Nanjing, China), following its instructions.

### Statistical analysis

All data were analyzed with GraphPad Prism 8.0 software and had been shown as the mean standard deviation (SD) or the standard error (SE) of three separate trials. The *t*-test or one-way analysis of variance (ANOVA) was used to determine the statistical significance between or among groups. Statistical significance was defined as *P* < 0.05.

## Results

### *Helicobacter pylori* upregulated circPGD expression in gastric cells and tissues

**Fig. 1:**
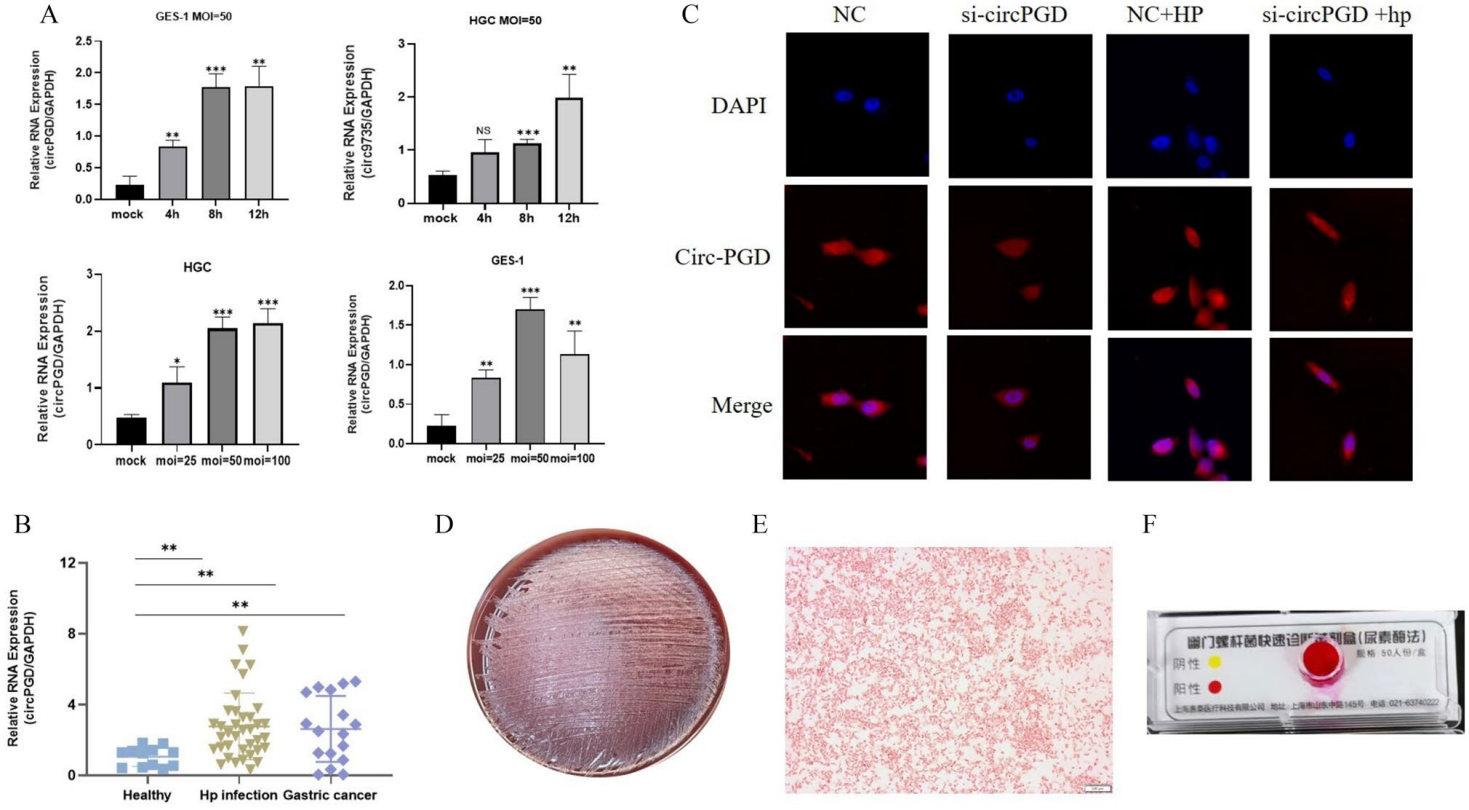
*Helicobacter pylori* upregulated circPGD expression in gastric cancer-associated cells and tissues. **A** The expression levels of circPGD were evaluated by qRT-PCR after *H. pylori* infection of cells. **B** The expression of circPGD in gastric tissues taken from healthy individuals, those with *H. pylori* infection, and patients with gastric cancer was examined using qRT-PCR.** C** FISH investigation circPGD expression and localization. **D** The colony morphology of *H. pylori* standard strain 26695 on Columbia blood plates. **E** Gram staining results of *H. pylori* strain 26695. **F** Urease test results of *H*. *pylori* strain 26695. *P < 0.05, **P < 0.01, ***P < 0.001

Using the human gastric mucosal epithelial cell line GES-1 and the human gastric cancer cell line HGC-27, we carried out infection studies to examine the effect of *H. pylori* on the expression of circPGD. *Helicobacter pylori* strain 26695 was introduced into these cell lines at varied multiplicities of infection (MOI) for varying durations, (25, 50, and 100 MOI; 4, 8, and 12 h). Then, the cellular circPGD expression levels were assessed. Notably, *H. pylori* 26695 infection dramatically increased the expression of circPGD at 50 and 100 MOI after 8 and 12 h of infection. At 50 MOI incubated for 8 h, this effect of increased expression stabilized (Fig. 1A). Consequently, the conditions of 50 MOI incubated for 8 h were used in all subsequent experiments. We also examined the expression of circPGD in gastric tissues obtained respectively from healthy individuals, those infected with *H. pylori*, and patients diagnosed with gastric cancer. The findings showed that circPGD expression in tissues of both *H. pylori*-infected individuals and gastric cancer patients was elevated when compared to the healthy controls (Fig. 1B). Moreover, we verified that *H. pylori* infection led to the enhanced expression of circPGD in both cytoplasm and nucleus of infected cells using fluorescence in situ hybridization (FISH) (Fig. 1C). Additionally, we evaluated the colony shape, Gram staining, and urease activity of the *H. pylori* strain 26695 used in our research (Fig. 1D–F).

### *Helicobacter pylori* administration reversed the previously observed reduction in migratory capacity and the epithelial-mesenchymal transition (EMT) process caused by circPGD knockdown

**Fig. 2:**
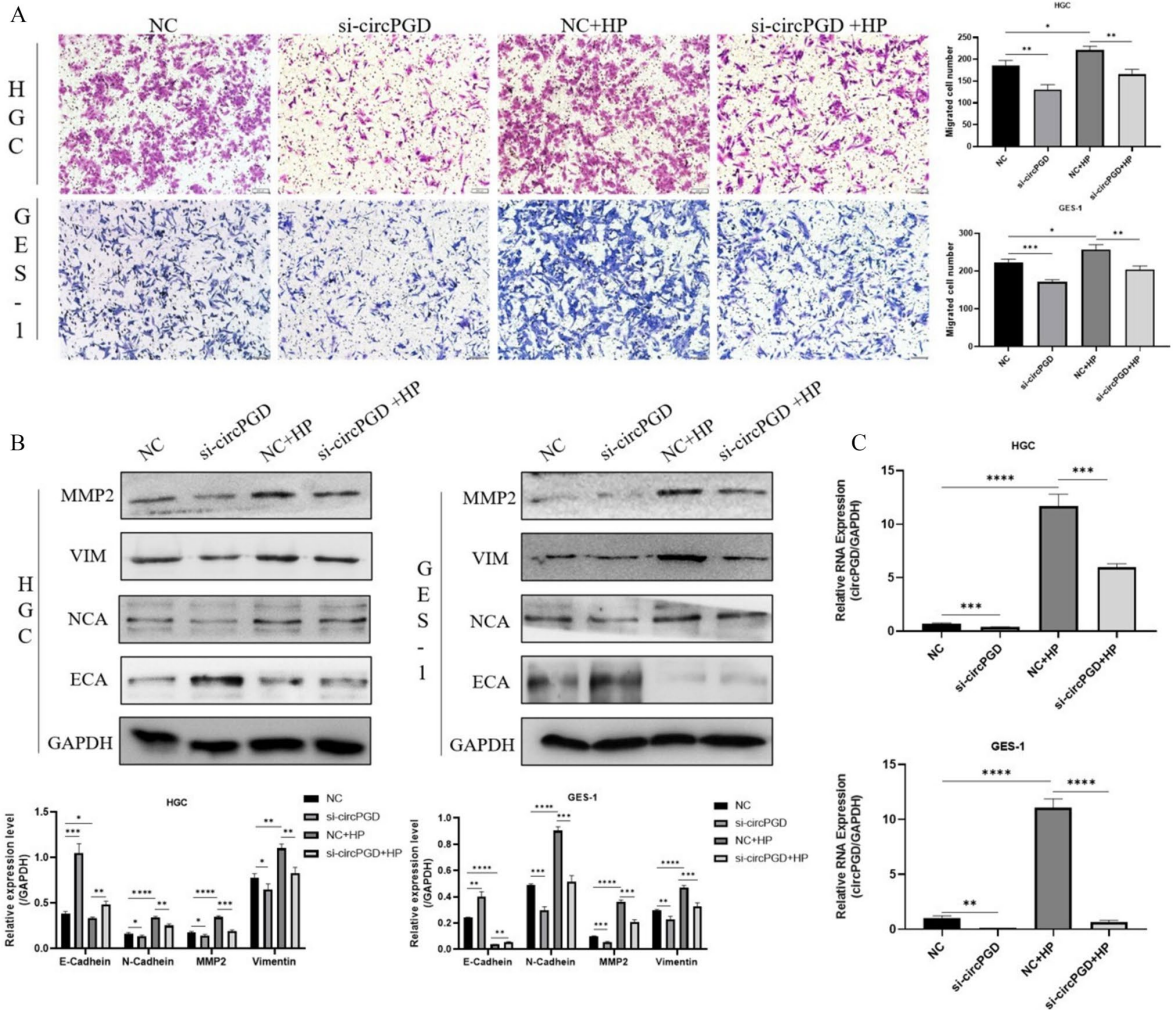
*Helicobacter pylori* reversed the reduced cell migratory capacity and the EMT process caused by circPGD knockdown. **A** The transwell assay results show that circPGD knockdown inhibited cell migration, while it was increased after *H. pylori* infection. **B** Western blotting results show that *H. pylori* altered the changes of protein expression levels of vimentin, N-cadherin, MMP2 and E-cadherin caused by circPGD knockdown. **C** The qRT-PCR results validated the efficiency of circPGD knockdown and indicate that circPGD expression was increased after *H. pylori* infection. *P < 0.05, **P < 0.01, ***P < 0.001

To investigate whether the increased expression of circPGD induced by *H. pylori* could promote cell carcinogenesis, we infected HGC-27 and GES-1 cells with *H. pylori* (50MOI, 8h) after transfecting them with siRNA to knock down circPGD. We found that *H. pylori* infection increased cell migration in the transwell migration tests, which was inhibited by the knockdown of circPGD (Fig. 2A). Furthermore, according to the Western blotting results, circPGD knockdown lowered the expression of proteins linked to EMT, including vimentin, N-cadherin, and MMP2, while increasing the expression of E-cadherin. It is interesting to note that *H. pylori* infection reversed the effects of circPGD knockdown, as shown by the increase in the expression of EMT-related proteins (Fig. 2B). According to the qRT-PCR results, circPGD expression decreased after circPGD knockdown and increased after *H. pylori* infection (Fig. 2C), supporting these findings.

### *Helicobacter pylori* further augmented the migratory capacity of cells and intensified the promotion of the EMT process caused by circPGD overexpression

**Fig. 3:**
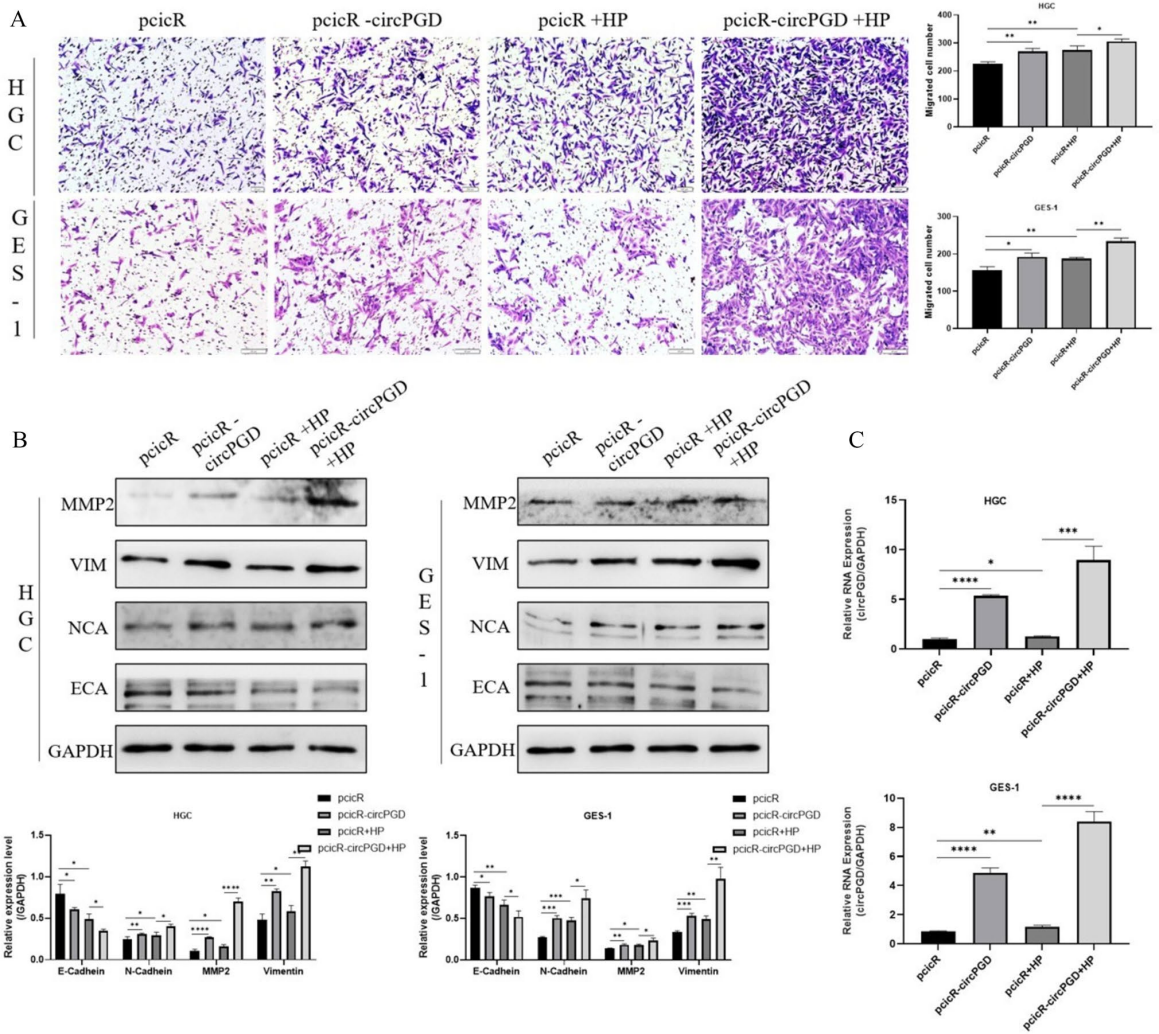
*Helicobacter pylor*i further enhanced the migratory ability of cells and increased the EMT process due to circPGD overexpression. **A** Vimentin, N-cadherin, MMP2, and E-cadherin levels changed in response to *H. pylori*, as detected by Western blotting. **B** The transwell assay showed that cell migration was improved by circPGD overexpression and was also enhanced after *H. pylori* infection (Fig. 3B). **C** The effective overexpression of circPGD was validated by qRT-PCR. *P < 0.05, **P < 0.01, ***P < 0.001

Because *H. pylori* was observed to promote cell carcinogenesis after circPGD knockdown, we infected HGC-27 and GES-1 cells with the pathogen after transfecting them with circPGD overexpression plasmids to further demonstrate the existence of a relationship between *H. pylori* and circPGD on the cancer-promoting process. We thus discovered that *H. pylori* infection facilitated cell migration, as indicated by the results of the transwell assay (Fig. 3A). Additionally, this impact was strengthened when cells were transfected with circPGD overexpression plasmids. Vimentin, N-cadherin, and MMP2 protein expression were all upregulated as a result of circPGD overexpression, whereas E-cadherin expression was downregulated, according to the Western blotting results. It should also be noted that *H. pylori* infection amplified these effects (Fig. 3B). Following transfection with the circPGD plasmid, the qRT-PCR analysis showed an increase in circPGD expression. It is also significant to note that *H. pylori* infection increased the expression of circPGD (Fig. 3C).

### *Helicobacter pylori* altered cell apoptosis and proliferation by modulating the expression of circPGD

**Fig. 4:**
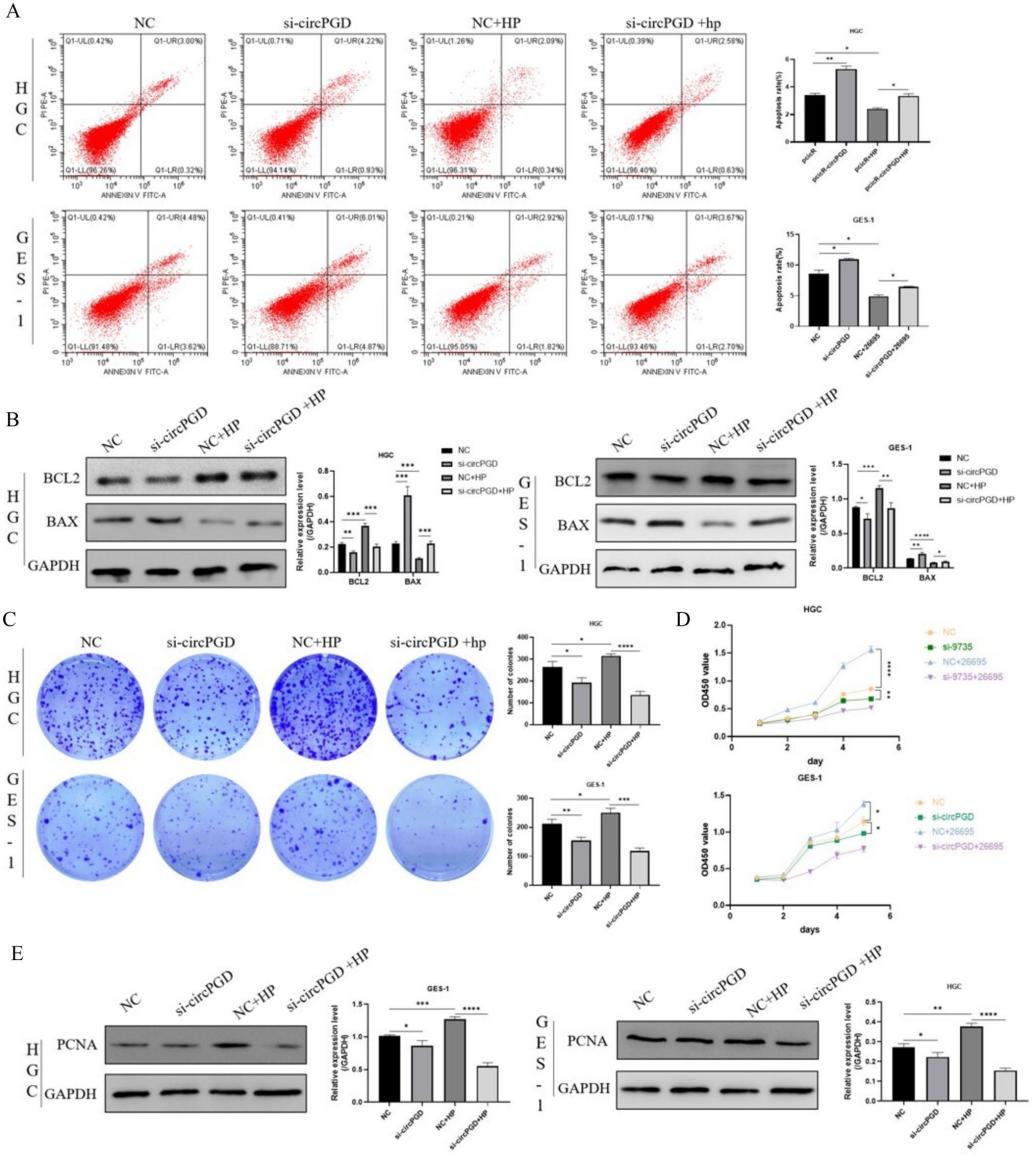
*Helicobacter pylori* affected cell apoptosis and proliferation by modulating the expression of circPGD. **A** Flow cytometry analysis of the impact of *H. pylori* infection and the silencing of intracellular circPGD on apoptosis. **B** Western blotting results showing that *H. pylori* infection increased PCNA expression in cells transfected with the negative control (NCs), but lowered its expression in the circPGD knockdown group. **C** Clone-formation experiments and **D** the CCK-8 assay results in contrast to the siRNA control group, showing that *H. pylori* increased cell proliferation, whereas circPGD knockdown reduced it. **E** PCNA expression was determined by Western blotting. *P < 0.05, **P < 0.01, ***P < 0.001

We performed tests in which circPGD was knocked down in HGC-27 and GES-1 cells, followed by *H*. *pylori* infection at MOI 50 for 8 h. Flow cytometry analysis revealed that *H. pylori* infection was associated with a decrease in the number of apoptotic cells, whereas the knockdown of circPGD resulted in a larger percentage of apoptotic cells compared to the control group (Fig. 4A). Western blotting analysis found that the knockdown of circPGD had the opposite effect to *H. pylori* infection, the latter of which upregulated Bcl-2 expression while downregulating BAX expression (Fig. 4B). Additionally, we used CCK-8 assay, clone-formation assay, and Western blotting to detect changes in cell proliferation capacity. The findings reveal that, in contrast to the siRNA control group, *H. pylori* increased cell proliferation, whereas circPGD downregulation reduced it. However, *H. pylori* attenuated the reducing effect of circPGD knockdown on cell proliferation (Fig. 4C, D). The Western blotting analysis of the cell growth marker protein PCNA produced consistent results (Fig. 4E). These results might have been influenced by the presence of additional regulatory elements or the reduced cell viability brought on by circPGD knockdown.

### *Helicobacter pylori* exacerbated the inhibitory effect of circPGD overexpression on apoptosis and promoted cell proliferation

**Fig. 5:**
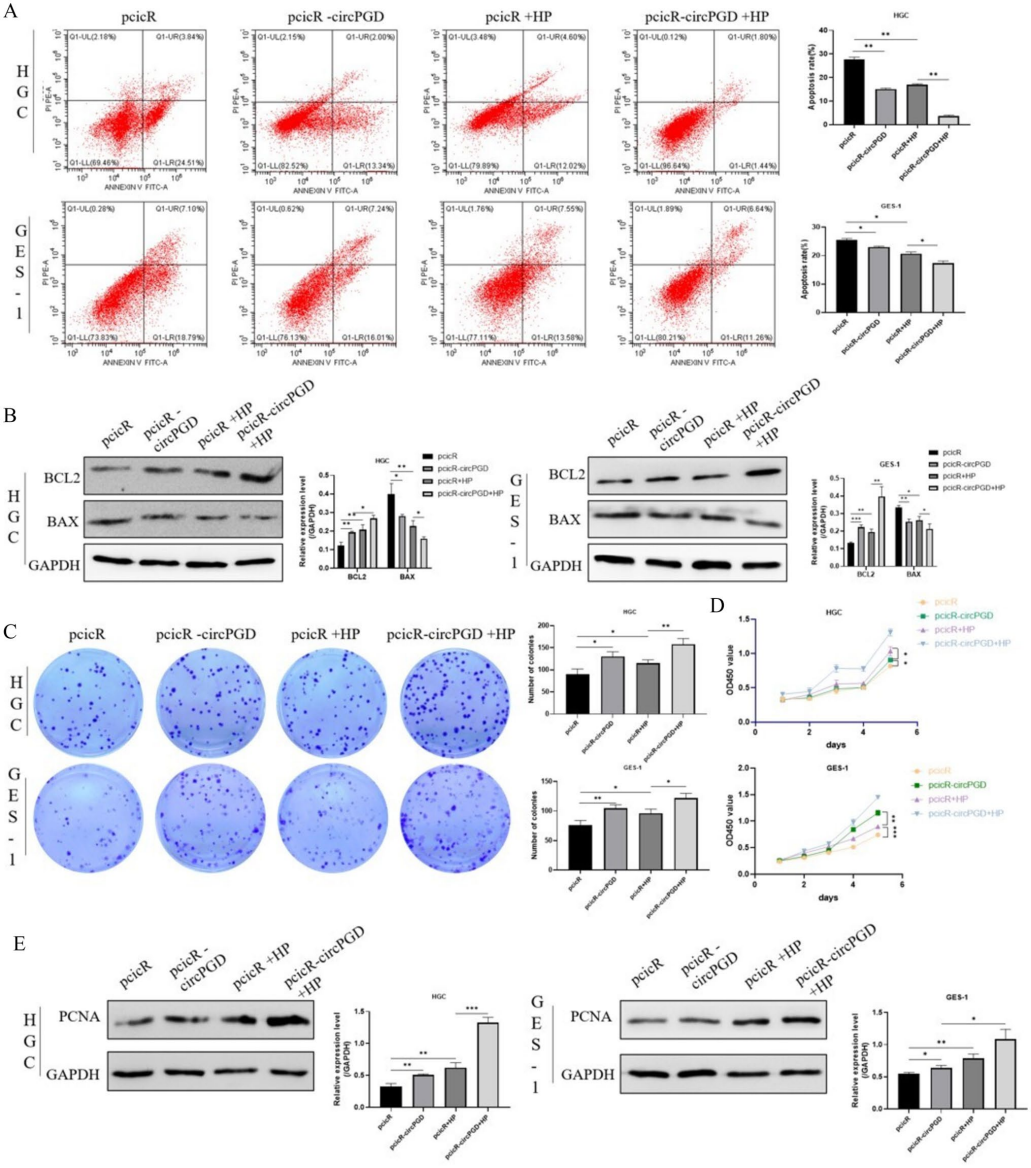
*Helicobacter pylori* exacerbated the inhibitory effect of circPGD overexpression on apoptosis and promoted cell proliferation. **A** Flow cytometry analysis of the impact of intracellular circPGD overexpression and *H. pylori* infection on apoptosis. **B** Western blotting was used to examine changes in the expression of proteins linked to apoptosis. **C** Clone-formation tests and **D** CCK-8 assay results, showing cell proliferation further facilitated by *H. pylori* infection. **E** PCNA expression was elevated by Western blotting. *P < 0.05, **P < 0.01, ***P < 0.001

*H. pylori* infection was established in HGC-27 and GES-1 cells following their transfection with circPGD overexpression plasmids. The circPGD overexpression group showed a reduced proportion of apoptotic cells after *H. pylori* infection compared to the control group, according to the flow cytometry analysis (Fig. 5A). Western blotting analysis showed that *H. pylori* infection decreased BAX expression and increased Bcl-2 expression (Fig. 5B). Through conducting Western blotting, CCK-8 test, and clone-formation assay, the changes in cell proliferation were evaluated (Fig. 5C–E). According to the findings, circPGD overexpression caused by *H. pylori* infection promoted cancer growth. These results further amplified *H. pylori* upregulated circPGD expression to prevent cell death and promote cancer growth.

### *Helicobacter pylori* exacerbated the inflammatory response in cells with circPGD knockdown

**Fig. 6:**
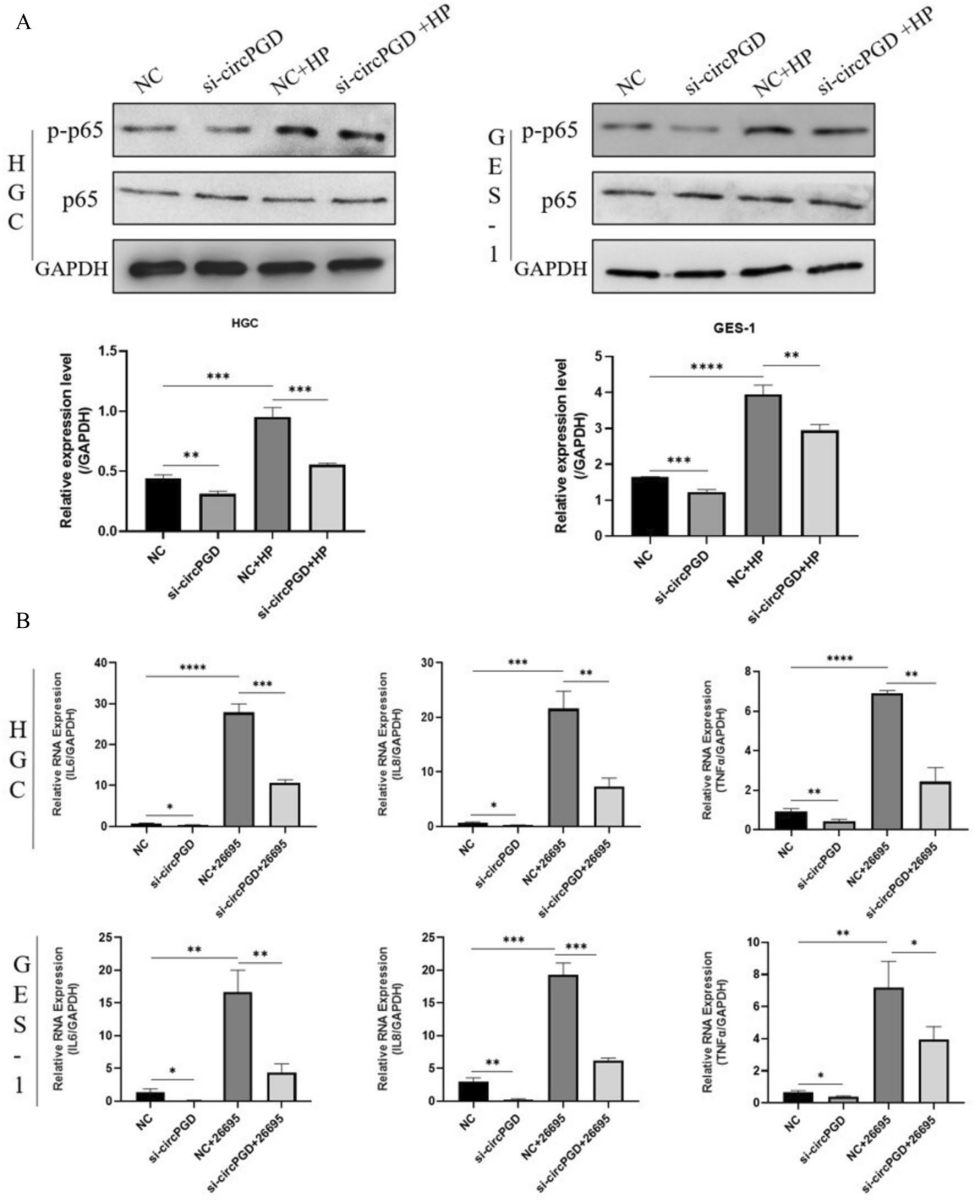
*Helicobacter pylori* enhanced the inflammatory response in cells with circPGD knockdown. **A** P-p65/p65 alterations were examined by Western blotting. **B** According to qRT-PCR, circPGD knockdown decreased the expression of markers linked to cellular inflammation, whereas *H. pylori* infection enhanced their expression

An enhanced inflammatory response is essential for the advancement of cancer attributed to *H. pylori* infection. HGC-27 and GES-1 cells were transfected with siRNA targeting circPGD and were then infected with *H. pylori*. As a consequence, Western blotting analysis showed that the phosphorylation of p65, a crucial protein in the NF-κB signaling pathway, was enhanced by *H. pylori* infection. In contrast to the control group, the inflammatory response was noticeably reduced in the circPGD knockdown group (Fig. 6A). Furthermore, qRT-PCR was used to measure the mRNA expression levels of the downstream inflammatory factors IL6, IL8, and TNFα. Our results show that all their expression levels were elevated as a result of *H. pylori* infection (Fig. 6B).

### *Helicobacter pylori* exacerbated the heightened inflammatory response induced by circPGD overexpression in cells

**Fig. 7:**
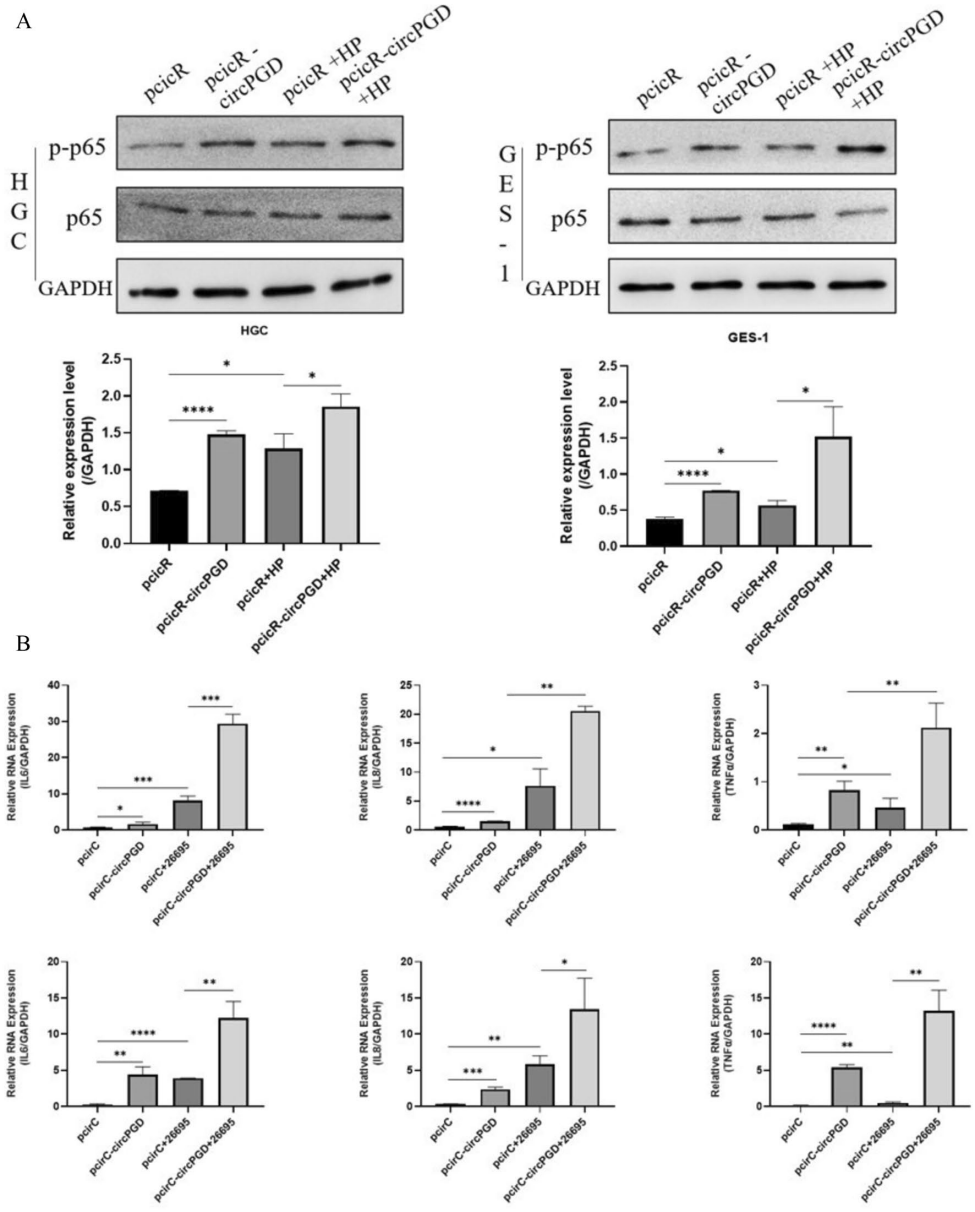
*Helicobacter pylori* further amplified the increased inflammatory response of cells caused by circPGD overexpression. **A** Western blotting was used to examine the changes in p-p65/p65 following circPGD overexpression and *H. pylori* infection. **B** According to qRT-PCR, circPGD overexpression increaseed the expression of markers associated with cellular inflammation, while *H. pylori* infection strengthened this effect

We used HGC-27 and GES-1 cells that had been transfected with circPGD overexpression plasmids before being infected with *H. pylori* to further study the ability of *H. pylori* to enhance the cellular inflammatory response mediated by circPGD. *H. pylori* infection enhanced the phosphorylation of p65, with greater phosphorylation levels being found in cells overexpressing circPGD in comparison to the control group (Fig. 7A). Additionally, qRT-PCR was used to measure the mRNA expression levels of IL6, IL8, and TNFα, finding that they were increased, especially in cells that overexpressed circPGD (Fig. 7B). These results jointly provide strong evidence that *H. pylori* can boost cellular inflammatory responses by triggering the increased expression of circPGD.

## Discussion

The death rate linked with gastric cancer remains disturbingly high, despite major scientific efforts in the field (Waldum and Fossmark 2021; McClain et al. 2017). This lack of progress is due in part to the intricate interplay between environmental, genetic, and immunological variables that contribute to the multifactorial development of gastric cancer (Suzuki et al. 2009; Cover et al. 2020). Our research team previously found that the chromosome 1 gene circPGD exhibited high expression levels in gastric cancer tissues, and furthermore, that its overexpression was associated with tumor size and lymph node metastasis in individuals with gastric cancer (Liu et al. 2022). Moreover, we showed that elevated circPGD expression promoted gastric cancer development by facilitating cell migration, proliferation, and EMT while it attenuated apoptosis. Building upon our earlier results, we wanted to better clarify how *H. pylori* participated in modulating circPGD as well as its influence on the development of gastric cancer. We used the *H. pylori* standard strain 26695 at different time points and MOI to infect the human gastric mucosal epithelial cell line GES-1 and the human gastric cancer cell line HGC-27. Our results show that *H. pylori* infection enhanced cellular circPGD expression, with the most prominent and sustained rise shown at 8 h and MOI = 50. Then we measured circPGD expression levels in gastric tissues taken from patients infected with *H. pylori* and those with gastric cancer compared to those with a healthy gastric profile by qRT-PCR, providing further evidence of the correlation between *H. pylori* and circPGD involvement in the occurrence and development of gastric cancer.

We performed Western blotting studies to measure the expression levels of vimentin, N-cadherin, E-cadherin, and MMP2, which are known to be related to carcinogenesis (Mentis et al. 2019; Shen et al. 2023) in order to learn more about the cellular processes driving it. Our findings reveal that *H. pylori* infection affected the expression of these proteins by increasing the expression of circPGD. Additionally, functional experiments, including cell migration assays, showed that *H. pylori* infection stimulated cell migration. This process could either be facilitated by overexpressing circPGD or inhibited by knocking it down. Together, our results offer convincing evidence that the *H. pylori*-induced elevation of circPGD promotes cell migration and the EMT processes, which in turn promote gastric carcinogenesis. We intended to explore changes in cell proliferation after *H. pylori* infection because aberrant cell proliferation is a defining characteristic of precancerous lesions (Yang et al. 2019; Ma et al. 2021), and because prior research has shown that elevated circPGD expression can enhance gastric cancer cell proliferation.

Apoptosis, which is a tightly controlled mechanism of programmed cell death, is essential for preserving cellular homeostasis and contributes to the development of cancer when dysregulated (Shen et al. 2022; Ghafouri-Fard et al. 2021; Fang et al. 2022). We measured the levels of Bcl-2 and BAX, two proteins of the Bcl-2 family of proteins (Yang et al. 2021), which are known to regulate apoptosis by regulating mitochondrial permeability (Xu et al. 2022; Li et al. 2020). Western blotting analysis was employed to decipher how *H. pylori* infection affects the expression of Bcl-2 and BAX. Our results show that *H. pylori* infection increased the expression of Bcl-2 and decreased that of BAX. Notably, circPGD knockdown consistently attenuated these alterations, while circPGD overexpression amplified the effects. These results were further supported by the results of the flow cytometry analysis, which showed a decrease in apoptosis following *H. pylori* infection. Together, these results offer strong proof that *H. pylori* infection inhibits apoptosis. This phenomenon can be attributed to the elevated expression of circPGD brought on by *H. pylori* infection. We also conducted CCK-8 and clone-formation assays (Wang et al. 2021; Tsukamoto et al. 2017), in addition to, Western blotting to measure PCNA protein expression, which is connected to proliferation. Our results showed that *H. pylori* infection stimulated cell proliferation while inhibiting the growth of cells with circPGD knockdown. Interestingly, the enhanced intracellular expression of circPGD was in opposition to this inhibitory effect. We speculate that other intracellular processes interact with the *H. pylori*-mediated control of cell proliferation to influence this phenomenon.

Significant geographical differences in the prevalence of gastric cancer have been identified by epidemiological studies (Xie et al. 2020). Notably, there is a clear link between the frequency of *H. pylori* infection and locations with the greatest incidence, especially in East Asia (Qin et al. 2022; Peng et al. 2022). Gastritis, which is viewed as a crucial stage in gastric carcinogenesis, is frequently present before the onset of gastric cancer (McClain et al. 2017; Watari et al. 2014). This process then advances through gastrointestinal chemosis, heterogeneous hyperplasia, and ultimately the development of cancerous lesions (Rihawi et al. 2021). In light of these findings, the goal of our current study was to look at changes in the molecular pathways involved in inflammation. We found that the overexpression of circPGD caused by *H. pylori* infection significantly increased the cellular inflammatory response, as evidenced by Western blot and qRT-PCR studies. These results highlight the part that elevated circPGD expression plays a role in escalating the inflammatory response and consequently aids the etiology of gastric cancer.

## Conclusion

In conclusion, our experimental results provide insights into the role of *H. pylori* in the regulation of circPGD expression, which subsequently results in a number of cellular processes linked to the advancement of gastric cancer. *H. pylori* infection induces the elevation of circPGD expression, further inhibits apoptosis, increases cell proliferation, migration and inflammatory response. These findings expand upon our current understanding of the underlying molecular processes driving *H. pylori*-related gastric cancer. The relevance of circPGD as a possible diagnostic marker and therapeutic target for gastric cancer is supported by our work. The results of our investigation have implications for improving existing clinical methods and creating tailored treatments for gastric cancer.

### Supplementary Information

Below is the link to the electronic supplementary material.

Supplementary file1 (DOCX 15 KB)

Supplementary file2 (DOCX 15 KB)

## Data Availability

All data generated or analysed during this study are included in this published article.
