# Supplementary material for: Helicobacter pylori upregulates circPGD and promotes development of gastric cancer
Source: J Cancer Res Clin Oncol. 2024 Feb 26;150(2):104. doi: 10.1007/s00432-023-05537-w (PMC10896836; doi:10.1007/s00432-023-05537-w)
Supplement: Supplementary file 1 — Supplementary file1 (DOCX 15 KB) [file 432_2023_5537_MOESM1_ESM.docx]

| Gene name | Sequence (5ʹ–3ʹ) |
| --- | --- |
|  | Forward: ACCATCTTCCAGGAGCGAGAT |
| GAPDH |  |
|  | Reverse:ATGACGAACATGGGGGCATC |
|  | Forward: ACATCAGACTGAGAGAAGCTGT |
| CircPGD |  |
|  | Reverse: GCGTAGAGTGCCTTCCGAAT |
|  | Forward: CACTGGTCTTTTGGAGTTTGAG |
| IL-6 |  |
|  | Reverse: GGACTTTTGTACTCATCTGCAC |
|  | Forward: GAATGGGTTTGCTAGAATGTGATA- |
| IL-8 |  |
|  | Reverse: CAGACTAGGGTTGCCAGATTTAAC |
|  | Forward: CCTCTCTCTAATCAGCCCTCTG |
| TNF-α |  |
|  | Reverse: GAGGACCTGGGAGTAGATGAG |
