# Supplementary material for: Helicobacter pylori upregulates circPGD and promotes development of gastric cancer
Source: J Cancer Res Clin Oncol. 2024 Feb 26;150(2):104. doi: 10.1007/s00432-023-05537-w (PMC10896836; doi:10.1007/s00432-023-05537-w)
Supplement: Supplementary file 2 — Supplementary file2 (DOCX 15 KB) [file 432_2023_5537_MOESM2_ESM.docx]

| Antibody name | corporate name |
| --- | --- |
| N-Cadherin antibody | Wanke Biotechnology Co., Ltd |
| E-Cadherin antibody | Wanke Biotechnology Co., Ltd |
| Vimentin monoclonal antibody | Protentech, USA |
| MMP2 monoclonal antibody | Protentech, USA |
| PCNA monoclonal antibody | Protentech, USA |
| Bcl-2 monoclonal antibody | Abcam, USA |
| Bax monoclonal antibody | Protentech, USA |
| GAPDH antibody | Abcam, USA |
| NF-κBp65 monoclonal antibody | Protentech, USA |
| p-NF-κBp65 monoclonal antibody | Abcam, USA |
